# The Hallmarks of Aging: From Molecular Mechanisms to Clinical Translation

**DOI:** 10.3390/ijms27188080

**Published:** 2026-09-11

**Authors:** Piotr Paweł Chmielewski, Bartłomiej Strzelec

**Affiliations:** 1 Division of Anatomy, Department of Human Morphology and Embryology, Faculty of Medicine, Wroclaw Medical University, 6a Chalubinskiego Street, 50-368 Wrocław, Poland; 2 University Center of General and Oncological Surgery, Wroclaw Medical University, 50-556 Wrocław, Poland

**Keywords:** aging, autophagy, biomarkers, geroscience, inflammaging, longevity, mitochondria, resilience, senescence, translation

## Abstract

Aging is a gradual process of structural and functional decline, marked by erosion of physiological integrity, adaptive capacity, and resilience, and by a concomitant increase in vulnerability to disease, disability, and death. The hallmarks of aging framework provides a widely used and experimentally tractable taxonomy of molecular and cellular processes associated with aging. In 2023, López-Otín and colleagues expanded the framework from nine to twelve hallmarks by adding disabled macroautophagy, chronic inflammation, and dysbiosis. Here, we evaluate the mechanistic and translational evidence for all twelve hallmarks. We also examine RNA-processing defects and extracellular matrix remodeling as candidate processes without classifying either as an additional hallmark. Experimental studies in model organisms support causal roles for several hallmarks. However, their causal priority, necessity, sufficiency, tissue specificity, and relevance to human aging remain unresolved. Clinical translation is limited by pleiotropy, compensatory responses, heterogeneous trajectories, uncertain biomarkers, and the long period required to detect meaningful outcomes. Future geroscience studies should treat hallmarks as provisional causal modules within interacting physiological networks, select mechanism-linked and function-centered endpoints, and test interventions against prospectively defined claims.

## 1. Introduction

**Figure 1:**
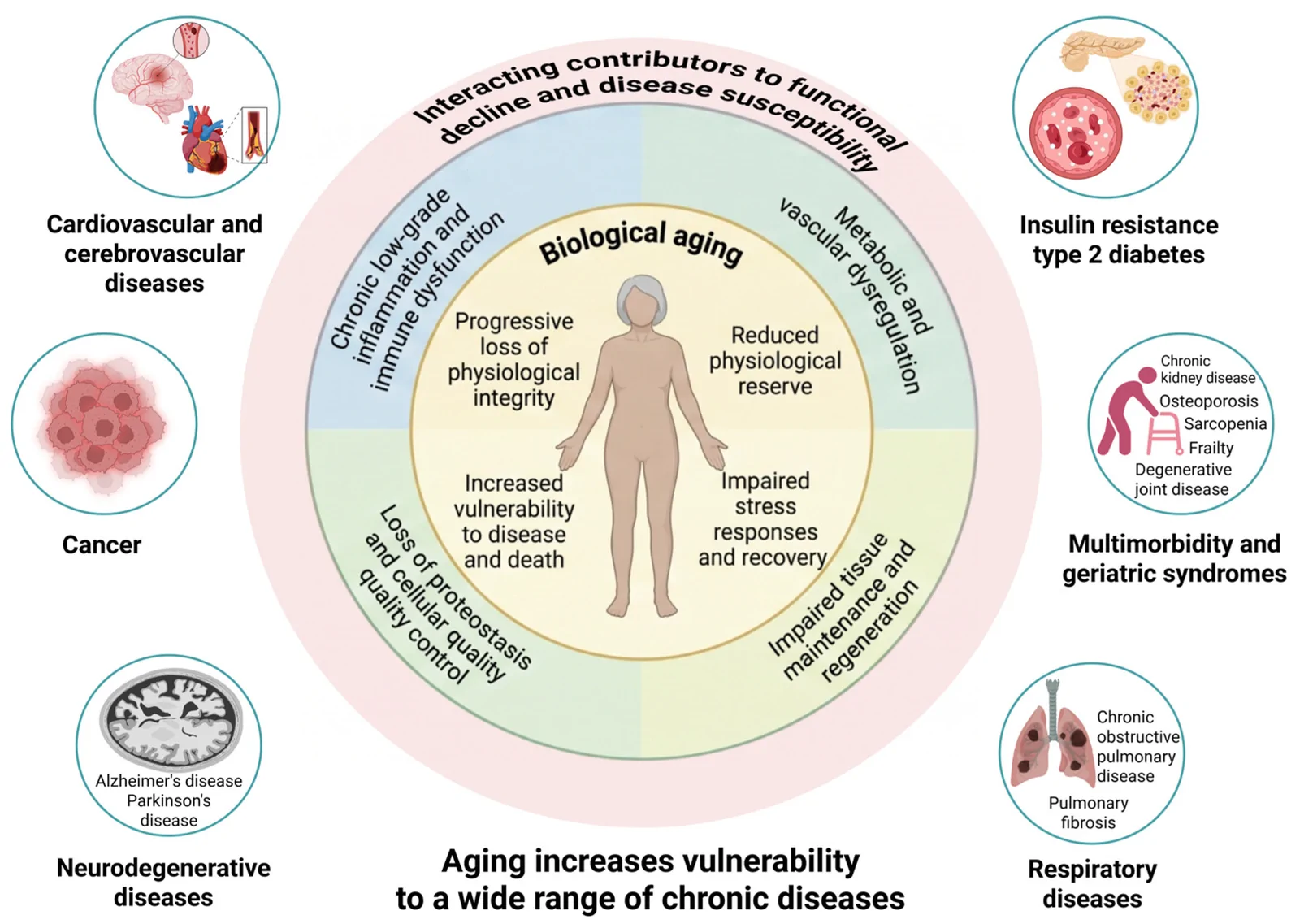
Biological aging, functional decline, and susceptibility to chronic disease. Aging entails the progressive loss of physiological integrity and reserve, impaired stress responses and recovery, and increasing vulnerability to disease, disability, and death. The figure depicts four interacting processes associated with functional decline and disease susceptibility: chronic inflammation and immune dysfunction, metabolic and vascular dysregulation, loss of cellular quality control and proteostasis, and impaired tissue maintenance and regeneration. These processes can increase susceptibility to cardiovascular and cerebrovascular diseases, cancer, neurodegenerative disorders, and metabolic dysfunctions, including type 2 diabetes. Their co-occurrence can contribute to multimorbidity and geriatric syndromes.

Population aging is increasing the prevalence of multimorbidity, frailty, disability, and care dependence [1]. Age is the strongest common risk factor for cardiovascular disease, type 2 diabetes, cancer, neurodegenerative disorders, and geriatric syndromes (Figure 1) [1,2]. The geroscience hypothesis posits that interventions directed at the processes implicated in biological aging could delay morbidity, preserve physiological function, and extend healthy lifespan. This proposition has motivated a shift from disease-specific treatment toward interventions that may affect multiple chronic conditions through shared biological processes [1,2,3,4,5,6]. Although broader benefits in older adults remain plausible, current evidence supports effects on specific diseases and functional outcomes.

Aging is not attributable to a single pathway and should not be construed as a uniform and necessary biological program [1,2,3,4,5,6,7,8]. Its manifestations vary across cell types, tissues, organ systems, individuals, and phases of the life course [9]. Genetic variation, sex, developmental history, infections, nutritional status, dietary patterns, physical activity, toxic exposures, psychosocial stress, socioeconomic status (SES), and access to medical care shape its pace and expression [7,8,9,10]. Such heterogeneity limits the expectation that targeting a single molecular pathway will produce uniform effects across individuals [4,7]. It also makes clinical translation dependent on baseline reserve, participant selection, exposure history, treatment timing, and competing risks [1,2,3,4].

**Figure 2:**
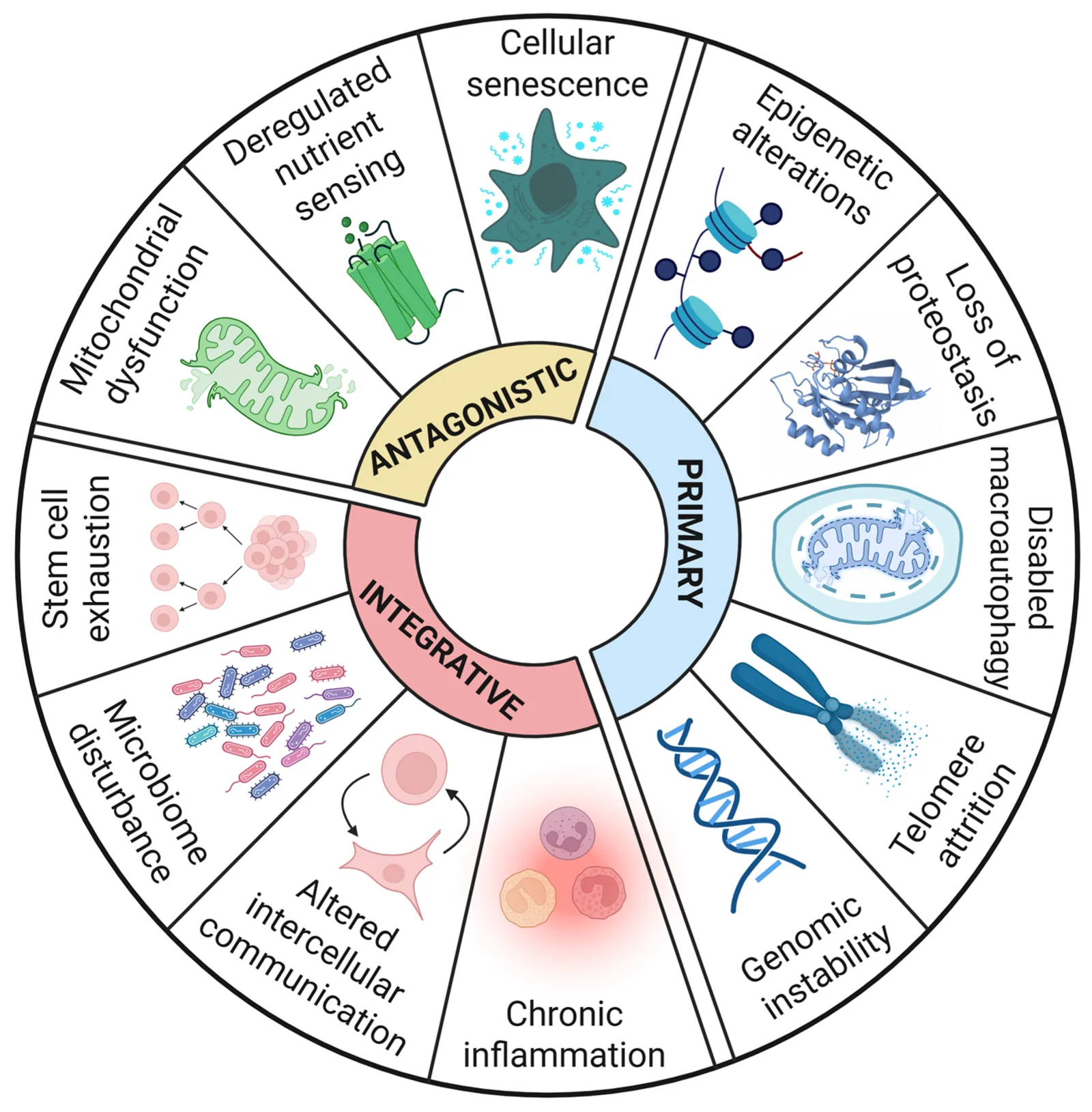
The twelve-hallmark framework of aging. The primary, antagonistic, and integrative categories are a heuristic classification and temporal tendency, not a fixed causal hierarchy. The primary hallmarks comprise processes considered to initiate molecular and cellular damage. The antagonistic hallmarks encompass context-dependent responses that can support adaptation or compensation when transient and appropriately regulated but become deleterious when chronic, excessive, or dysregulated. The integrative hallmarks emerge when accumulated damage and prolonged responses exceed homeostatic and regenerative capacity, contributing to stem cell exhaustion, altered intercellular communication, chronic inflammation, dysbiosis, and functional decline. Feedback is bidirectional across categories: an integrative state such as chronic inflammation can act upstream of mitochondrial, proteostatic, metabolic, and stem-cell dysfunction.

The hallmarks of aging framework, first formulated by López-Otín et al. in 2013 [2] and expanded by López-Otín et al. in 2023 [11], organizes recurrent molecular and cellular processes into twelve interconnected hallmarks: genomic instability, telomere attrition, epigenetic alterations, loss of proteostasis, disabled macroautophagy, deregulated nutrient sensing, mitochondrial dysfunction, cellular senescence, stem cell exhaustion, altered intercellular communication, chronic inflammation, and dysbiosis (Figure 2). The framework has supplied a common language for experiments, biomarker programs, and therapeutic development [4,6,11,12,13,14]. The hallmarks overlap, operate at different levels of biological organization, and differ in causal evidence, measurability, reversibility, and proximity to clinical outcomes [7,8,11]. Some describe sources or manifestations of damage, some encompass initially adaptive responses, and others capture integrative states [6,8,11]. Whereas the 2023 *Cell* review updated the taxonomy and synthesized its mechanistic basis, the present review adds a four-question appraisal of each hallmark, operationalizes the concept of a provisional causal module, distinguishes levels of translational inference, and links molecular perturbation to function, resilience, and clinically testable claims.

We propose a framework for evaluating the hallmarks based on four key questions. First, does the process change reproducibly with age in relevant human tissues? Second, does experimental manipulation alter age-related function or survival rather than an isolated molecular readout? Third, is the effect conserved across biological contexts? Fourth, can the process be measured and modified in humans? We apply this framework to illustrate strengths and limitations in the current evidence base, examine interactions among the hallmarks, consider candidate additions, and outline requirements for clinical translation. We argue that the hallmarks are most useful when treated as provisional causal modules embedded in networks that maintain homeostasis and resilience [4,6,7,8,11,12,13,14,15,16,17,18,19,20,21,22,23,24,25,26,27,28,29,30].

## 2. Genomic Instability

Nuclear and mitochondrial genomes are continuously exposed to replication errors, spontaneous hydrolysis, reactive metabolites, ultraviolet (UV) and ionizing radiation, environmental factors, and inflammatory oxidants [30,31]. Base-excision repair, nucleotide-excision repair, mismatch repair, homologous recombination, nonhomologous end joining, and ATM/ATR-dependent DNA-damage responses preserve genomic function, but none is error-free [30,31,32]. With age, somatic mutations, structural variants, chromosomal abnormalities, clonal expansions, and persistent DNA-damage foci accumulate in tissue-specific patterns [30,33,34,35,36]. Single-cell sequencing shows that mutation burden rises across diverse human tissues, whereas clonal hematopoiesis and mutant epithelial clones illustrate how selection acts on this variation [33,34,35]. Biological consequences depend less on mutation count alone than on genomic location, cellular context, clonal behavior, and damage-induced signaling [30,36]. Defects in genome-maintenance genes cause segmental progeroid syndromes, reduced DNA-repair capacity can shorten lifespan in experimental models, and long-lived species can exhibit enhanced genome maintenance [31,37].

Nevertheless, these findings do not establish the accumulation of somatic mutations during life as a sufficient explanation for aging in humans without defined genome-maintenance disorders [30,31]. Many mutations are neutral, and cells can tolerate substantial mosaicism [30,35,36]. Persistent damage signaling may be as consequential as altered DNA sequence [20,30,32]. ATM, ATR, p53, and PARP pathways can induce cell-cycle arrest, senescence, apoptosis, metabolic remodeling, or inflammatory signaling. Cytosolic chromatin fragments and derepressed retroelements activate cGAS-STING and type I interferon programs, linking genome instability to inflammation [20,21,22,38]. These responses protect against malignant transformation but can propagate tissue dysfunction, illustrating the tension between short-term protection and late-life cost [20,30].

8-Oxoguanine DNA glycosylase 1 (OGG1) is a base-excision-repair enzyme that excises the oxidative lesion 8-oxoguanine. The experimental small-molecule activator TH10785 increased OGG1 catalytic activity and conferred β,δ-lyase activity, thereby accelerating oxidative-lesion repair in cells [39]. OGG1 can also be targeted in the opposite direction. The small-molecule inhibitor TH5487 prevents OGG1 from binding 8-oxoguanine in gene promoters and suppresses proinflammatory gene expression, and the same compound reduces myofibroblast transition, inflammatory cell recruitment, and collagen deposition in a murine model of pulmonary fibrosis [40,41]. A single enzyme can therefore be modulated toward opposite ends, and the appropriate direction depends on whether repair capacity or OGG1-dependent transcriptional signaling is the intended target. This is evidence of pharmacological target modulation, not of slowed organismal aging; organism-level efficacy and long-term safety remain unresolved.

Translation requires separating repair enhancement from generalized suppression of DNA-damage responses. Increasing repair fidelity or reducing genotoxic exposure is conceptually attractive, but checkpoint attenuation could permit survival and expansion of oncogenic cells [30,31]. Interventions directed at nucleotide metabolism, NAD^+^-dependent repair, sirtuins, transposable elements, or cytosolic DNA sensing may affect several hallmarks concurrently [20,21,22,38]. Their evaluation should include cancer surveillance, clonal dynamics, tissue-specific mutation spectra, and functional outcomes. Genome-maintenance disorders establish clinical actionability for specific lesions, but they do not show that the same interventions modify aging in people without those disorders.

## 3. Telomere Attrition

Telomeres protect chromosome ends through TTAGGG repeats, shelterin, T-loop architecture, and telomeric chromatin [42,43,44]. In most somatic cells, incomplete end replication and limited telomerase activity shorten telomeres with cell division, while oxidative lesions and replication stress can accelerate telomere attrition. Critically short or uncapped telomeres activate persistent ATM- or ATR-dependent DNA-damage signaling, which promotes p53–p21-mediated arrest, senescence, apoptosis, and altered tissue repair. Telomere dysfunction thereby connects genome maintenance, stem cell reserve, senescence, and inflammation. It is most consequential in proliferative compartments, although telomere-associated paracrine and systemic signaling can affect postmitotic tissues [44].

Human telomere-biology disorders provide strong evidence that short or dysfunctional telomeres can cause pulmonary fibrosis, bone-marrow failure, liver disease, and immunodeficiency [45]. In mice, telomerase deficiency causes progressive tissue degeneration, whereas telomerase reactivation or gene transfer can restore function in selected settings [46,47,48,49,50]. Interpretation requires caution because laboratory mice have substantially longer telomeres than humans, and telomerase-deficient models represent severe telomere failure rather than age-associated telomere dynamics in wild-type animals. Leukocyte telomere length is associated with several age-associated outcomes at the population level, but measurement variability, leukocyte-composition effects, inherited differences, reverse causation, and limited individual discrimination restrict its use as a universal aging biomarker [51,52]. Attrition rate may be informative, but repeated measurement adds substantial error [42,51].

Therapeutic approaches include treatment of defined telomere syndromes, reduction in oxidative and inflammatory stress, and cautious attempts at transient telomerase activation [44,50,52]. In a prospective study of telomere-biology disorders, orally administered danazol was associated with hematologic responses and reduced telomere attrition, although its androgenic and other pleiotropic actions preclude interpretation as selective telomerase activation [53]. Preclinical approaches include systemic adeno-associated viral delivery of telomerase reverse transcriptase (AAV-TERT) in adult or old mice and in mouse models of pulmonary fibrosis [48,50]. Candidate telomerase activators should not be presented as established geroprotectors. The central liability is cancer because short telomeres constrain proliferation and can suppress early tumor growth, whereas dysfunctional telomeres can promote chromosomal instability [43,44]. Telomerase reactivation may restore regenerative capacity but can support malignant clones. Translation should therefore prioritize genetically or clinically defined telomere failure, use time-limited or tissue-directed delivery, and combine telomere endpoints with clonal and oncological surveillance. A slower decline in leukocyte telomere length can support a mechanistic claim, but cannot alone establish slower aging [42,51,52].

## 4. Epigenetic Alterations

Age alters DNA methylation, histone modifications, nucleosome positioning, chromatin accessibility, three-dimensional genome organization, transposable-element control, and noncoding RNA programs [21,22,38,54,55,56,57,58]. These changes include stochastic changes and coordinated responses to DNA damage, inflammation, metabolism, cell turnover, and shifts in cell composition [55,56,58]. Loss of H3K9me3-rich heterochromatin and derepression of repetitive elements can disrupt transcriptional homeostasis and activate innate immune signaling [21,22,38]. DNMT and TET enzymes, histone acetyltransferases and deacetylases, chromatin remodelers, and metabolites including acetyl-CoA, S-adenosylmethionine, NAD^+^, and α-ketoglutarate connect epigenetic regulation to nutrient sensing, mitochondrial function, and DNA repair [54,55,56,57,58,59,60,61]. Thus, epigenetic alterations are both readouts and potential mediators of aging-related dysfunction.

DNA-methylation clocks provide reproducible predictors of chronological age, mortality risk, or physiological state [55,56,62,63,64,65,66]. First-generation clocks were trained primarily on chronological age, whereas later models incorporated mortality-associated proteins, smoking exposure, clinical phenotypes, causal information, longitudinal change, or cross-species conservation [56,62,63]. These tools do not measure a common biological entity because chronological-age predictors, mortality-derived clocks, and mitotic clocks encode different information [55,56,62]. Cell composition, ancestry, cohort structure, and acute physiological stress can affect estimates [55,62,63,64,67]. Changes after pregnancy, surgery, infection, or exercise show that some clock outputs are dynamic, but biomarker reversibility is not equivalent to reversal of organismal aging [64,65,66].

Experimental manipulation supports a causal contribution from some chromatin regulators [54,59,60]. Partial reprogramming with OCT4, SOX2, and KLF4, with or without MYC, can reset selected molecular features and improve vision, regeneration, or function in mice [68,69,70,71,72,73]. The risks include loss of cell identity, dysplasia, teratoma formation, oncogenesis, and incomplete control of tissue-specific responses [11,69,73]. In the cited study, a two-vector AAV9 system delivered a doxycycline-inducible OCT4-SOX2-KLF4 (OSK) cassette systemically to 124-week-old male C57BL/6J mice. Treatment increased median remaining lifespan and improved a frailty index in this single preclinical experiment [68]. Because only male mice were studied, the experiment did not determine whether the effects differ by sex. No clinical study has established the safety or efficacy of systemic partial reprogramming in humans, and the long-term risks of loss of cell identity, dysplasia, and oncogenesis remain unresolved. Human studies reporting changes in epigenetic age remain exploratory and do not establish durable clinical benefit [5,18,19,62].

For intervention trials, epigenetic clocks should be treated as candidate response biomarkers [5,18,19,62]. A useful clock should be analytically reliable, responsive in the relevant tissue or an explicitly justified proxy, mechanistically linked to the intervention, and predictive of clinically meaningful outcomes beyond conventional risk factors [5,18,19]. Concordance across clocks is desirable but insufficient because correlated algorithms can share training biases [18,62]. Trials should prespecify the clock, expected direction and magnitude of change, sampling interval, cell-composition adjustment, and relationship to function. The strongest inference will come from concordant changes in mechanism-linked biomarkers, physiological reserve, and clinical outcomes.

## 5. Loss of Proteostasis

Proteostasis integrates protein synthesis, folding, trafficking, compartmentalization, repair, and degradation [26,74,75,76,77,78,79,80,81,82]. Molecular chaperones, the ubiquitin–proteasome system, endoplasmic-reticulum and mitochondrial unfolded-protein responses, macroautophagy, chaperone-mediated autophagy, and stress-granule dynamics cooperate to maintain a functional proteome. Aging reduces the capacity or coordination of several components, permitting oxidized, glycated, misfolded, and aggregation-prone proteins to accumulate [76,82]. Translation fidelity and elongation kinetics also change with age, linking ribosome function directly to proteome quality [76,83,84,85]. Proteostasis cannot be inferred from aggregate abundance alone. The unfolded protein response and integrated stress response can restore homeostasis when transient, whereas prolonged eIF2α-ATF4 signaling can suppress translation, remodel metabolism, and promote dysfunction [75,86]. Aggregate burden reflects the balance among synthesis, folding, sequestration, and clearance, and aggregates may represent toxic species, protective deposits, or markers of upstream failure [74,76,87].

Genetic enhancement of chaperone activity, proteasome function, translation fidelity, chaperone-mediated autophagy, or selective macroautophagy extends lifespan or preserves tissue function in model organisms of aging [74,78,82,83,84,85,88,89,90,91,92]. Chemical chaperones and modulators of the unfolded protein response have been evaluated in aged mice and in defined proteotoxic disorders, with indication-specific and mixed efficacy [80,81]. Translation is constrained by pathway pleiotropy. Chronic suppression of protein synthesis can impair growth and repair, sustained stress signaling can become maladaptive, and enhanced proteostasis can support malignant-cell survival [26,27,74,75]. Therefore, interventions should identify the defective proteostasis step, demonstrate restored flux or fidelity, and monitor tissue repair, immunity, and cancer.

## 6. Disabled Macroautophagy

Macroautophagy is an evolutionarily conserved lysosomal degradation pathway that sequesters cytoplasmic cargo within double-membrane autophagosomes and delivers it to lysosomes via autophagosome–lysosome fusion for degradation and recycling [26,27,87]. Its core machinery includes ULK1-complex activation, class III PI3K-VPS34-dependent phagophore nucleation, ATG12-ATG5-ATG16L1 conjugation, LC3/GABARAP lipidation, cargo-receptor engagement, and HOPS- and SNARE-mediated autophagosome–lysosome fusion. TFEB-family transcription factors coordinate lysosomal biogenesis and autophagy-related gene expression. Selective pathways remove mitochondria, protein aggregates, lipid droplets, pathogens, and damaged organelle domains [93]. Because autophagosome formation and lysosomal degradation can change independently, autophagic flux, rather than a static LC3-II or p62 measurement, is the mechanistically relevant quantity [77].

Loss of core autophagy genes causes severe tissue degeneration, whereas *Atg5* overexpression or disruption of the beclin 1–BCL-2 interaction extends lifespan and preserves function in mice [88,89,90]. Caloric restriction, mTORC1 inhibition, exercise, and spermidine can stimulate autophagy, although each affects many other pathways [10,27,91]. Urolithin A can engage mitophagy-related programs and has produced selected functional or biomarker effects in human trials, but it has not been shown to modify human aging [93,94]. Human studies of NAD^+^ precursors show biochemical target engagement with inconsistent functional benefit [95,96,97]. These data support tractability, not equivalence among autophagy induction, mitophagy, and organismal rejuvenation [27,87,93].

The therapeutic window is context-dependent. Autophagy can suppress tumor initiation by limiting cellular damage, yet established tumors can use it to survive hypoxia, nutrient restriction, and treatment [26,27]. Increasing autophagosome biogenesis will not restore clearance when lysosomal acidification, hydrolase activity, trafficking, or membrane fusion is limiting [87]. Mitophagy also requires coordinated mitochondrial fission, cargo recognition, and lysosomal competence [93]. Accordingly, clinical studies must identify the rate-limiting defect, quantify pathway flux in the relevant tissue, and prespecify oncological and immunological safety.

## 7. Deregulated Nutrient Sensing

Nutrient-sensing networks couple substrate availability to growth, reproduction, stress resistance, and maintenance [10,11]. Insulin and IGF-1 activate PI3K-AKT signaling, which inhibits FOXO factors and promotes anabolic programs, whereas amino acids and growth factors activate mTORC1. AMPK responds to cellular energy deficit, suppresses mTORC1, and promotes catabolic maintenance, while sirtuins link NAD^+^ availability to deacylation of metabolic, chromatin, and stress-response proteins [10,11,97]. Longevity effects of reduced IIS or mTOR signaling vary by pathway branch, tissue, sex, dose, developmental stage, and nutritional state [98].

Caloric restriction without malnutrition extends lifespan in several species and remodels insulin-IGF-1, mTOR, AMPK, autophagy, circadian, and inflammatory pathways [3,10,99,100]. In humans, moderate caloric restriction produces cardiometabolic and immune changes, but evidence for longer lifespan is unavailable. Meal timing, protein and amino-acid composition, adiposity, and circadian alignment can modify the response. Benefits cannot be assigned to one nutrient-sensing node, and restriction can be harmful in frail, sarcopenic, or undernourished individuals. Intervention trials should therefore distinguish dietary pattern, energy deficit, weight loss, and molecular target engagement.

Rapamycin and related mTOR inhibitors extend lifespan in mice, and transient or low-dose regimens can preserve selected functions [101,102]. Low-dose TORC1 inhibition increased interferon-stimulated antiviral gene expression and reduced laboratory-confirmed respiratory infections in a phase 2b study. However, a phase 3 trial did not reduce clinically symptomatic respiratory illness [103,104]. These divergent outcomes illustrate the dependence of geroscience trials on population selection and endpoint definition. Metformin is being evaluated as a geroscience intervention because it alters energy metabolism and disease risk, although evidence that it slows human aging is absent [105]. Acarbose extends lifespan in mice with marked sex dependence, underscoring the need to model heterogeneous treatment effects [106]. NAD^+^ precursors produce consistent biochemical changes but variable clinical outcomes [95,96,97]. These interventions test distinct components of nutrient sensing and should not be treated as interchangeable geroprotectors [3,10,11].

## 8. Mitochondrial Dysfunction

Mitochondria integrate ATP production, redox control, biosynthesis, calcium handling, apoptosis, innate immune signaling, and metabolite-dependent regulation of chromatin [11,23,97,107,108,109,110,111,112,113]. Aging can alter respiratory-chain function, membrane potential, mitochondrial dynamics, mtDNA integrity, and organelle turnover, but direction and magnitude vary among tissues and physiological states [107,108,109,112]. Reactive oxygen species (ROS) are signaling molecules as well as sources of molecular damage [110]. Low or transient mitochondrial stress can induce adaptive mitohormetic responses. Conversely, sustained or excessive stress promotes damage and dysfunction. This dose dependence helps explain why indiscriminate antioxidant supplementation has not reproduced the effects predicted by a simple free-radical theory.

Mitochondrial quality depends on coordinated fission, fusion, biogenesis, proteostasis, and mitophagy, including PINK1–Parkin-dependent and receptor-mediated pathways [23,93]. Damaged mitochondria can release mtDNA, cardiolipin, formyl peptides, and reactive species that activate cGAS-STING, Toll-like receptors, and inflammasomes. The resulting inflammatory output depends on the route and amount of mtDNA release, the receiving cell, and countervailing mitophagy. Somatic mtDNA mutations expand clonally in some human tissues, but their causal importance varies, and total mutation burden does not directly predict functional decline [107,108,109,110,111,112].

Exercise remains the best-established intervention for improving mitochondrial capacity and whole-body function [3,10]. Pharmacological candidates include urolithin A, NAD^+^ precursors, and modulators of mitochondrial dynamics or quality control [93,94,95,96,97]. Human studies have shown target engagement and selected metabolic or functional effects, but results are heterogeneous and do not establish slower organismal aging [94,95,96,97]. A mitochondrial intervention should be evaluated using tissue-appropriate bioenergetics, mtDNA and quality-control markers, and functional outcomes while accounting for sex, training status, and baseline metabolic disease [5,93,97,107]. These outcomes support pathway-specific efficacy and should not be extrapolated to organism-level modification of aging.

## 9. Cellular Senescence

Cellular senescence is a heterogeneous, context-dependent state of stable cell-cycle arrest in metabolically active cells, typically triggered by telomere dysfunction, oncogenic signaling, genotoxic or oxidative stress, mitochondrial dysfunction, chromatin disruption, or cytotoxic therapy. Senescent cells often acquire a senescence-associated secretory phenotype (SASP) and display features such as increased senescence-associated β-galactosidase (SA-β-gal) activity [24,114]. This durable arrest is distinct from quiescence and terminal differentiation, although rare escape from senescence or re-entry into the cell cycle can occur in selected experimental settings or in some neoplastic contexts [24]. The p53-p21CIP1 and p16INK4a-RB axes commonly enforce arrest, whereas p38 MAPK, mTOR, NF-κB, C/EBPβ, JAK-STAT, and cGAS–STING shape additional features whose expression varies with the initiating stress, cell lineage, tissue, and time [24,114]. Transient senescence contributes to embryogenesis, tissue remodeling, wound repair, and tumor suppression [24]. Adverse effects arise chiefly when senescent cells persist because their formation exceeds immune clearance, or when their local signaling becomes chronic [24,114].

Direct quantification is difficult because no single senescence marker is specific or universal. Nevertheless, telomere-dysfunction-associated foci in dermal fibroblasts of aging primates and p16INK4a expression in human peripheral-blood T cells increase strongly with age [115,116]. These observations support accumulation of defined senescence-associated cell states in particular tissues and cell populations, but they do not establish a uniform organism-wide burden.

A persistent DNA damage response (DDR) is common, but not universal, in senescent cells [24]. ATM-CHK2 and ATR-CHK1 signaling can stabilize p53 and p21CIP1, while unresolved lesions form γH2AX- and 53BP1-positive foci, telomere-associated foci, or DNA segments with chromatin alterations that reinforce senescence [24]. Cytoplasmic chromatin fragments and mtDNA can activate cGAS-STING, which connects genome or organelle damage to NF-κB-dependent inflammation [24,114]. Senescent or damaged cells can also release or expose alarmins, including HMGB1, extracellular ATP, and DNA-containing material, which engage pattern-recognition receptors in neighboring stromal and immune cells [24,114]. None of these features is specific. Reliable identification therefore requires a combination of proliferative arrest, expression of p16INK4a or p21CIP1, SA-β-gal accumulation and activity, lipofuscin or lysosomal expansion, loss of lamin B1, persistent DNA-damage foci, and secretory markers [24].

The SASP is a dynamic and nonuniform mixture of cytokines, chemokines, growth factors, matrix-remodeling proteases, bioactive lipids, extracellular vesicles, and other mediators [24,114]. Common inflammatory components include IL-6, CXCL8/IL-8, CCL2, CXCL1, IL-1α, and, in selected contexts, IL-1β and TNF [24,114]. Surface-associated IL-1α can activate IL-1R–IRAK1 signaling and NF-κB and C/EBPβ, which places it upstream of the IL-6-CXCL8 network in several senescence models [117]. IL-1β maturation instead requires inflammasome-caspase-1 activity and is not a universal SASP component [24,114]. The SASP can reinforce arrest, recruit immune-mediated clearance, coordinate repair, or suppress tumors [24,114]. Conversely, persistent SASP output can transmit paracrine senescence, alter the extracellular matrix and stem-cell niches, impair endothelial and metabolic function, and sustain myeloid activation [24,114]. These local and circulating signals can contribute to chronic systemic inflammation, although their quantitative contribution in humans remains tissue- and disease-dependent [24,114].

Senolytics selectively induce death in senescent cells by exploiting senescent-cell anti-apoptotic pathways, including BCL-2-family, PI3K-AKT, SRC-family, ephrin-receptor, p53, and HSP90 dependencies [114]. The most studied senolytics include dasatinib plus quercetin (D+Q), fisetin, and navitoclax [114]. Navitoclax has substantial preclinical activity but dose-limiting thrombocytopenia, whereas the cellular selectivity and pharmacology of D+Q and fisetin vary across tissues and senescence inducers [114]. Combined suppression of cellular FLICE-like inhibitory protein (cFLIP) and activation of death receptor 5 (DR5) sensitized therapy-induced senescent cancer cells to senolysis [118]. Because this evidence derives from cancer models, it is an oncology-specific proof of principle rather than validation of DR5 agonists as a general senolytic class for aging. Genetic clearance of p16INK4a-positive cells, D+Q, and fisetin improve selected diseases, physical function, or survival in mice [119,120,121]. Small human studies of D+Q in idiopathic pulmonary fibrosis and diabetic kidney disease established feasibility and reported preliminary functional or tissue-biomarker signals [122,123].

In a randomized phase 2 trial in postmenopausal women, D+Q did not reduce the primary bone-resorption endpoint in the full cohort. Exploratory responses were concentrated among women with higher baseline senescent-cell burden [124]. Senomorphics suppress selected SASP components without eliminating the cell [114]. Rapamycin, metformin, JAK inhibitors, and modulators of p38 MAPK or NF-κB show such activity in experimental systems, but their pleiotropy precludes attribution of human effects specifically to SASP inhibition [114]. No senolytic or senomorphic has yet been shown to slow human aging [114,124]. Trials require tissue-relevant evidence of target engagement, multidimensional senescence markers, clinically meaningful endpoints, and surveillance for impaired repair, infection, hematological toxicity, and cancer [24,114,124].

## 10. Stem Cell Exhaustion

Tissue maintenance depends on resident stem and progenitor cells, facultative plasticity, supportive niches, and systemic signals [125]. Aging can alter stem cell abundance, quiescence, self-renewal, lineage output, and injury responses, but patterns differ sharply among tissues. Hematopoietic stem cells expand numerically while showing myeloid bias, clonal selection, and impaired regenerative function, whereas muscle satellite cells and neural progenitors lose activation or differentiation competence [34,35,125]. Epithelial tissues may retain turnover while accumulating mutant clones. Telomere dysfunction, epigenetic drift, mitochondrial dysfunction, impaired proteostasis, inflammation, and changes in extracellular matrix (ECM) can contribute through cell-autonomous and niche-dependent mechanisms [11,78,90,125,126].

Stem cell exhaustion is an integrative outcome rather than a primary lesion [11,125]. Restoration of autophagy, metabolic regulation, or niche signaling can improve stem-cell function and tissue regeneration in mice [90,125]. Such strategies carry distinct risks. Expansion can favor oncogenic clones, whereas transplantation may replace a deficient compartment without correcting the aged niche or systemic environment [125]. Clinical translation should define whether the goal is increased cell number, improved lineage output, restored injury response, or durable organ function. Measures of clonal diversity, mutation burden, and malignancy are essential whenever regenerative capacity is increased.

## 11. Altered Intercellular Communication

Aging cannot be inferred from cell-autonomous changes alone [11,127]. Neural, endocrine, immune, vascular, and paracrine signals coordinate metabolism, stress responses, repair, and behavior [11,126,127,128,129,130]. Aging alters insulin and IGF-1 signaling, sex steroids, adrenergic and renin–angiotensin systems, myokines, adipokines, hepatokines, complement, and coagulation [11]. Extracellular vesicles (EVs) transfer proteins, lipids, mRNAs, and regulatory RNAs, while gap junctions, innervation, ECM, and vascular perfusion provide additional routes of communication [11,126,128]. Increased signaling noise and loss of spatial or temporal coordination can be as consequential as altered mean concentrations [7,127]. Heterochronic parabiosis and blood-exchange experiments show that circulating environments can rapidly affect tissue function in mice [128,131,132,133].

Nevertheless, these studies do not justify unregulated use of young plasma in humans. Dilution of circulating factors, albumin replacement, immune effects, and procedural variables complicate interpretation [131,132]. Candidate pro-aging factors include CCL11, β2-microglobulin, complement components, TGF-β, and inflammatory cytokines, but none explains systemic aging alone [11,128]. Defined circulating cytokines and tissue-specific trophic factors can improve selected functions in old mice [129,130]. Translation should target defined signals or physiological systems rather than poorly characterized plasma products [4,128,129,130,131,132,133].

## 12. Chronic Inflammation

The term “inflammaging” refers to chronic, low-grade inflammatory activity that develops in many older adults and is associated with frailty, disability, cardiovascular events, cancer, and increased morbidity and mortality [25,134,135,136,137]. It is heterogeneous in its sources, cellular composition, anatomical distribution, and temporal course [25,137]. Its sources include senescent cells, visceral adiposity, damaged mitochondria, cytosolic DNA, impaired autophagy, barrier failure, periodontal disease, tissue fibrosis, and environmental exposures [25,134,135,136,137]. Immunosenescence, including impaired adaptive responses, altered myeloid function, clonal hematopoiesis, and defective resolution, can coexist with chronic innate activation. The resulting state reflects dysregulated timing, localization, magnitude, and resolution rather than a uniform increase in immunity [25,137].

Pattern-recognition receptors and downstream NF-κB, JAK-STAT, cGAS-STING, and NLRP3 pathways connect upstream damage to inflammatory gene expression and cytokine maturation [25,136,137]. NLRP3 activation requires transcriptional priming and a second signal that promotes inflammasome assembly, caspase-1 activation, and maturation of IL-1β and IL-18 [136,137]. CANTOS showed that IL-1β inhibition reduces recurrent cardiovascular events in selected patients with residual inflammatory risk but increases fatal infection [138]. This finding establishes therapeutic causality in a defined disease context, not that systemic cytokine blockade slows aging [138]. Anti-inflammatory treatment may remove a harmful driver, suppress an adaptive response, or do both, which makes participant selection and infection surveillance essential [25,138].

Regular exercise, smoking cessation, treatment of obesity, adequate sleep, and nutritionally sufficient diets can reduce inflammatory burden while improving several physiological systems [1,3,25,137]. The clinical benefits do not need to be assigned to one hallmark [1,3]. Pharmacological approaches include cytokine antagonists, inflammasome inhibitors, senotherapeutics (senolytics that selectively eliminate senescent cells and senomorphics that attenuate the SASP), mTOR modulators, and drugs that alter metabolism and immune function [136,137,138]. Trials should distinguish inflammatory biomarkers from immune competence and measure vaccine response, infection, wound repair, and functional recovery. Therefore, chronic inflammation is best understood as a network state integrating damage, failed clearance, barrier dysfunction, and altered intercellular communication.

## 13. Dysbiosis and Barrier Dysfunction

**Figure 3:**
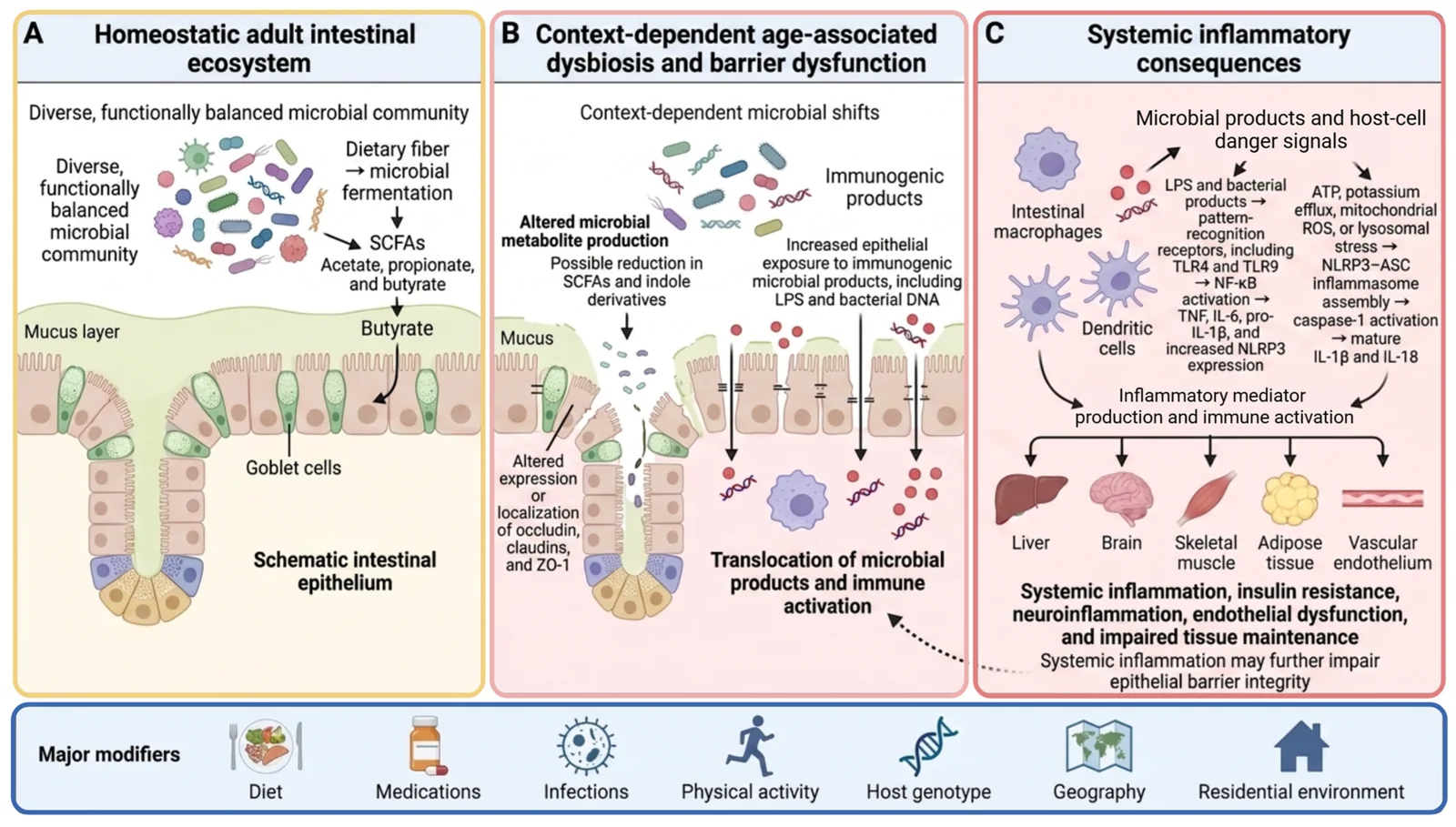
Gut dysbiosis, barrier dysfunction, and systemic inflammation during aging. (**A**) In intestinal homeostasis, gut microorganisms convert dietary fiber into SCFAs that support epithelial integrity and immune function. (**B**) Context-dependent age-associated microbial shifts can alter metabolite production, weaken mucus and tight junctions, and increase microbial-product translocation. Microbial products prime NF-κB-dependent inflammatory transcription, whereas host–cell danger signals can promote NLRP3 assembly, caspase-1 activation, and IL-1β and IL-18 maturation. (**C**) Resulting inflammation can impair metabolic, neural, muscular, and vascular function and further weaken the intestinal barrier. However, diet, medications, infections, physical activity, host genotype, geography, and residential environment can modify these relationships.

The gut microbiome contributes to nutrient processing, SCFA production, bile-acid transformation, epithelial integrity, immune education, and xenobiotic metabolism [139,140,141]. Later life is frequently associated with changes in community structure, ecological stability, and microbial metabolites, but trajectories vary markedly among individuals and populations [140,141]. Diets, medications, disease, and environmental exposures can shape these patterns. A universal aged microbiome has not been identified to date, and functional output might be more informative than mere taxonomic diversity or composition. Animal-transfer studies support causality in selected contexts. Microbiota from young donors can improve immune, reproductive, behavioral, or survival-related outcomes in aged or progeroid recipients [142,143,144,145]. In contrast, human evidence is less conclusive because disease, medications, and altered physiology can themselves induce gut dysbiosis (Figure 3), and microbial shifts may reflect compensation rather than causation [140,141,146]. Loss of mucus or tight-junction integrity can increase exposure to LPS, bacterial DNA, and other microbial products, thereby engaging pattern-recognition receptors and systemic inflammatory signaling [146]. Microbiome effects also extend beyond the gut through SCFAs, secondary bile acids, tryptophan metabolites, trimethylamine-related pathways, vagal signaling, and immune modulation [139,140,147].

Dietary fiber, prebiotics, probiotics, postbiotics, and fecal microbiota transplantation (FMT) can modify microbial functions or community structure [140,141,148]. Nonetheless, efficacy is established for a limited set of indications, not for aging itself. Responses vary with baseline ecology, diet, medication, host genotype, and donor characteristics. Safety concerns include pathogen transmission, antimicrobial-resistance genes, and unpredictable metabolic effects. Geroscience trials should define the microbial function to be modified, verify the relevant metabolite or barrier pathway, and connect it to host physiology. Although the microbiome is modifiable, biological complexity makes generalized rejuvenation claims untenable.

## 14. Candidate Processes and Omitted Dimensions

No finite list can capture every age-related process. RNA-processing defects and extracellular-matrix (ECM) remodeling are considered here as candidate processes, not established additions to the twelve-hallmark framework [11,149]. RNA-processing defects are plausible because alternative splicing, intron retention, RNA modifications, RNA surveillance, and ribonucleoprotein homeostasis vary across tissues and can generate aberrant isoforms that burden proteostasis [150,151].

Some splicing factors can affect lifespan in model organisms, and splice-switching oligonucleotides establish therapeutic tractability in defined genetic diseases [150]. However, evidence that generalized correction of RNA processing modifies mammalian aging remains limited. RNA dysregulation may be partly downstream of genomic, epigenomic, and proteostatic defects. Its incremental explanatory value is post-transcriptional: isoform choice, RNA surveillance, and ribonucleoprotein homeostasis can be perturbed even when DNA sequence and chromatin state do not identify the relevant defect. Separate-module status would require prediction or intervention effects beyond those provided by genomic, epigenomic, and proteostatic measurements. ECM remodeling and altered tissue mechanics are insufficiently represented by the current taxonomy [2,11,152,153,154]. ECM adds a more distinct tissue-level dimension by storing damage in long-lived structural components, shaping mechanotransduction, and constraining regenerative niches. Collagen crosslinking, elastin fragmentation, glycation, fibrosis, and altered matrix turnover affect vascular compliance, lung elasticity, muscle function, neural plasticity, and stem cell niches. Cells sense stiffness through integrins, focal adhesions, YAP-TAZ, TGF-β, WNT, and related pathways, creating feedback among matrix damage, senescence, inflammation, and regenerative failure [126,154,155].

Because many matrix proteins are long-lived, damage can retain a cumulative record of exposure that is not rapidly corrected by cell replacement [154]. Interventions against glycation, fibrosis, crosslinks, or mechanosignaling require tissue-specific evaluation because ECM remodeling is also necessary for repair [154,155]. Impaired RNA homeostasis, loss of cytoskeletal integrity, and altered mechanical properties can be framed as candidate modules or as dimensions of existing hallmarks [11,12,149,150,151,154,155]. Accordingly, the choice should depend on explanatory and experimental value rather than the frequency with which a process appears in age-associated datasets [8,12]. A candidate process should offer causal insight, a validated measure, and an intervention strategy that improves outcomes relevant to aging and lifespan [8,12].

## 15. From Descriptive Hallmarks to Causal Modules

The original framework proposed three criteria for a hallmark: age-associated manifestation, experimental aggravation that accelerates aging, and experimental amelioration that delays or attenuates aging [2,11]. These criteria are operationally sound. However, they are insufficient unless aging and the relevant outcome are defined explicitly. A change may accompany age because it is causal, compensatory, or consequential. Experimental aggravation can produce pathology without reproducing aging, whereas improving a molecular abnormality can relieve a disease phenotype without altering the wider process that generates multisystem vulnerability. 

Strong causal inference requires temporal ordering, dose–response relationships, reversibility where biologically plausible, mediation, replication across models, and convergence among genetic, pharmacological, and environmental evidence [5,7]. Human relevance also requires evidence that the same mechanism operates within clinically accessible ranges [4,5,6,15,16,17,18]. A favorable inflammatory marker or epigenetic-clock response does not establish delayed disability or mortality [5,18,19]. A disease-specific benefit can reduce multimorbidity but might create liabilities in other tissues [3,17]. Therefore, mechanistic and clinical outcomes must be aligned prospectively, with claims restricted to the level directly tested [15,16,17,18].

A provisional causal module is defined operationally as a bounded, experimentally tractable set of interacting processes for which perturbation changes one or more prespecified age-relevant functional outcomes through an identifiable mechanism. This definition requires more than association or prediction. An age-associated biomarker may signal a process without mediating it, a downstream manifestation may change without affecting upstream determinants, and a compensatory response may be beneficial or harmful depending on biological context and time. Causal independence is neither expected nor required. A process warrants separate-module status when its definition supports measurements or interventions that add explanatory, predictive, or experimental value beyond existing modules. Integrative hallmarks can be analyzed as modules when a tractable mechanism can be isolated within the broader phenotype, but the phenotype should not be treated as causally uniform. Causal position is conditional on tissue, life stage, challenge, and network state. The transition from adaptation to dysfunction depends on challenge magnitude, duration and recurrence, baseline physiological reserve, regenerative capacity, efficiency of damage clearance, termination of the stress response, and completeness of recovery. These determinants might explain why the same process, e.g., mitochondrial stress, cellular senescence, autophagy, or inflammation, can be protective when transient yet harmful when persistent or poorly resolved.

The division of the hallmarks into primary, antagonistic, and integrative categories is accordingly a heuristic classification and a temporal tendency rather than a fixed causal hierarchy, because feedback operates in both directions across the three categories. The hallmarks can be modeled as partially overlapping modules connected by feedback, compensation, and resource allocation [7,11]. For example, DNA damage can induce senescence and inflammatory signaling, whereas inflammation can impair stem cell function, mitochondrial quality, insulin sensitivity, and proteostasis [20,21,22,23,24,25]. Mitochondrial dysfunction can activate innate immunity via mtDNA release, while autophagy can restrain or intensify this response depending on lysosomal competence and cargo flux [23,24,25,26,27]. Such reciprocal relations predict nonlinear responses and make the effect of targeting one node dependent on the state of the network [7,28]. Combination therapy may be required in some contexts, but indiscriminate multitarget treatment can increase toxicity and obscure causal interpretation [28,29]. Rational combinations should be based on demonstrated mechanistic interaction, nonredundant benefit, compatible pharmacokinetics and pharmacodynamics, and prospectively specified safety criteria. Table 1 summarizes the evidence available in the literature for all twelve hallmarks and identifies the main limits on causal and translational inference.

## 16. Clinical Translation

Translation begins with a clearly defined claim [15,16,17]. Target engagement asks whether an intervention changes its intended molecular process. Biomarker response asks whether a prespecified indicator changes in a direction linked to the mechanism. Disease modification asks whether the intervention alters the course of a defined disease. Functional preservation asks whether it maintains mobility, cognition, sensory function, or independence. Resilience asks whether an organism can respond to a challenge and recover from it. Modification of organismal aging is the broadest claim and requires sustained benefit across multiple domains, with acceptable safety. These levels are neither automatically sequential nor substitutable: evidence at a lower level does not establish benefit at a higher level, and each transition requires its own design and endpoint. Consequently, a single molecular surrogate cannot establish modification of organismal aging [7,16,17,18]. Trials should specify which level they are testing and should not retroactively upgrade the claim after a favorable biomarker result.

Static state measures characterize resting status, whereas dynamic resilience measures quantify a response trajectory after a standardized perturbation. Relevant features include peak displacement, coordination across systems, response duration, time to recovery, residual deficit, and repeatability. Exercise, vaccination, surgery, and metabolic challenges can therefore reveal differences between individuals with similar resting biomarkers. This challenge-response-recovery framework connects the earlier distinction between transient adaptation and persistent dysfunction to clinically measurable physiological reserve.

Biomarkers should be selected according to their context of use [5,16,18,19,156,157,158]. Diagnostic biomarkers identify a state; prognostic biomarkers predict an outcome; predictive biomarkers identify likely treatment response; pharmacodynamic biomarkers show biological activity; and surrogate endpoints can replace clinical outcomes only after stringent validation. Most proposed aging biomarkers are prognostic or pharmacodynamic rather than validated surrogates. Composite omics clocks might improve prediction, but their biological meaning and sensitivity to intervention vary. Function-focused measures, including gait speed, handgrip strength, cardiorespiratory fitness, cognition, frailty indices, and recovery after vaccination, exercise, surgery, or metabolic challenge, provide complementary information [16,156,157,158,159,160,161].

A therapy aimed at senescence may require evidence of senescent-cell burden, whereas a telomere intervention should focus on telomere dysfunction rather than chronological age alone. A mitochondrial intervention may be most informative in participants with impaired oxidative capacity [15,16,17,18]. Moreover, stratification should consider sex, frailty, multimorbidity, medication, and baseline reserve. Intermittent treatment may be appropriate for pathways in which chronic inhibition impairs repair, immunity, or metabolism. Follow-up should be long enough to detect delayed harms, especially cancer, infection, and loss of tissue function. At minimum, clinically persuasive evidence should include target engagement in the relevant tissue or a justified proxy, changes in mechanism-linked biomarkers, improvement in a prespecified functional or disease outcome, durability of benefit, and acceptable safety. Claims of modifying organismal aging in healthy humans require evidence of benefit across multiple physiological domains and reduced vulnerability, rather than improvement in an isolated measure of performance [4,7,16]. In later-phase trials, multimorbidity incidence, disability-free survival, and validated composite clinical outcomes provide stronger endpoints than unvalidated estimates of molecular age.

A staged translational program can reduce overinterpretation and unnecessary exposure. Early studies should establish dose, safety, target engagement, and a mechanism-linked response in an appropriate tissue or justified proxy. Mechanism-enriched randomized phase 2 trials should then test a prespecified disease, functional, or challenge-recovery endpoint. Only interventions with concordant benefit and acceptable safety should proceed to longer trials of multidomain function, multimorbidity, disability-free survival, or other outcomes capable of supporting an organismal-aging claim [15,16,17,18].

Precision geroscience and medicine should not mean indiscriminate personalization based on large biomarker panels [4,5,7,16], but should match an intervention to a causal mechanism that is measurable and modifiable in a defined individual or group, and then test whether the predicted downstream benefit occurs [4,17,18]. This approach accommodates different biological routes to similar functional decline and allows negative findings to refine the underlying model [4,17]. If target engagement does not improve function, the target may be downstream, redundant, engaged at the wrong time, or relevant only to a particular group of patients [5,17].

## 17. Conclusions and Perspectives

The hallmarks of aging framework organizes molecular and cellular processes associated with aging into an experimentally tractable scheme, but it neither defines a unique causal hierarchy nor demonstrates that modifying any single pathway or target alters biological aging as a whole. Progress now depends on resolving interactions, causal priority, tissue specificity, and human relevance through longitudinal studies, single-cell and spatial analyses, physiological testing, and mechanism-informed clinical trials. Because aging emerges from interacting processes across all levels of biological organization, individual mechanisms should be evaluated within the compensatory systems that shape them. Therefore, the hallmarks framework should guide testable, systems-level hypotheses rather than function as a checklist of drug targets, and its clinical translation requires valid measurements, defined populations, and prospectively specified molecular and functional outcomes that are meaningful to older adults.

## Figures and Tables

**Table 1:** Evidence base for the twelve hallmarks of aging: experimental support, human relevance, measurements, and translational status.

Hallmark	Age-Associated Evidence	Experimental Evidence	Major Modifiers	Measurement and Translation
**Genomic** **instability**	Reproducible accumulation of somatic mutations, clonal mosaicism, chromosomal alterations, and persistent DNA damage signaling across human tissues [30,33,34,35]	Defects in genome-maintenance genes cause progeroid syndromes [31]; comparative and human genetic evidence links SIRT6-associated genome maintenance to longevity [37,60,61]	Lesion type and location, clonal dynamics, tissue context, trade-offs with tumor suppression [30,31]	Somatic mutations, once fixed, are irreversible, so intervention must be preventive; no approach that enhances genome maintenance has been tested in humans against somatic mutation accumulation [31]
**Telomere** **attrition**	Reproducible telomere shortening with replicative age in proliferative human tissues; dysfunction correlates with replicative senescence and tissue exhaustion [42,43,44]	Loss-of-function mutations in telomerase and telomere maintenance genes cause premature tissue failure [45]; telomerase reactivation reverses degeneration in telomerase-deficient mice [46,47,48]	Species-specific telomere biology, inherited length, cellular turnover rate, oxidative and inflammatory stress, trade-offs with cancer risk [42,43,49]	Danazol slows leukocyte telomere attrition in telomere biology disorders, on evidence from a small, uncontrolled trial [53]; gene therapy is efficacious in mice, but remains preclinical [48,50]
**Epigenetic** **alterations**	DNA-methylation and chromatin changes, partly attributable to cell composition and exposures [55,58,67]	Chromatin manipulation and partial reprogramming can improve selected functions in mice [54,69,70,73]	Tissue, cell composition, exposure, dose, and timing [55,67]	Epigenetic clocks are widely used predictors of age-related outcomes, but they are not validated surrogate endpoints [18,62,63]; partial reprogramming and epigenetic “rejuvenation” are being investigated [69,70]
**Loss of** **proteostasis**	Impaired folding, degradation, and translational fidelity, with aggregate accumulation [74,76,85]	Enhanced quality control preserves function or lifespan in model organisms [82,83,92]	Stress duration, cargo, cellular state, and tissue [74,75]	Measurement is problematic, as no validated human assay of proteostatic capacity exists [74]
**Disabled** **macroautophagy**	Reduced autophagic flux and lysosomal competence. Static markers are inconclusive [27,87]	Autophagy-gene and selective-autophagy manipulations improve function and extend lifespan in animal models [88,89,90,91]	Cargo, lysosomal capacity, timing, cancer, and infection [26,93]	Dynamic flux is difficult to assess, particularly in human tissues [27]; fine-tuning is necessary as crude enhancement is not desired, given the roles of autophagy in tumor cell survival and immunity [26]
**Deregulated** **nutrient sensing**	Insulin, IGF-1, mTOR, and AMPK signaling shift with age, adiposity, and disease status, though not uniformly across tissues. In humans, nutrient-sensing tone is inferred from downstream metabolic and immune readouts rather than measured directly [10,11,100]	Reduced GH/IIS signaling, caloric restriction, and mTORC1 inhibition can extend lifespan in animal models, and transient rapamycin treatment can do so in mice [98,99,100,101,102]	Sex, genotype, dietary composition as distinct from calorie amount, the timing and duration of fasting, and dose intermittency, since continuous and intermittent mTORC1 inhibition yield different immune and metabolic outcomes [99,103,106]	No validated human measure of nutrient-sensing tone exists. Two years of caloric restriction in non-obese adults altered immune and metabolic markers [100]; repurposed drugs (e.g., rapamycin, metformin) are being tested
**Mitochondrial** **dysfunction**	Respiratory capacity, mitochondrial dynamics, and mitophagy decline heterogeneously across human tissues. Somatic mtDNA point mutations and deletions can undergo clonal expansion in selected postmitotic and stem-cell compartments; functional consequences generally emerge when mutant load exceeds a tissue- and mutation-specific threshold [107,108,109,112]	Restoration of mitochondrial quality control improves function in mice, whereas mild mitochondrial stress can extend lifespan through mitohormesis [11,93,110]; mtDNA released into the cytosol activates cGAS–STING and inflammasome signaling [23]. T cells bearing dysfunctional mitochondria are sufficient to induce premature multimorbidity in mice [134]	Heteroplasmy threshold and clonality, tissue energetic demand and proliferative status, the presence of a physiological challenge, and stimulus dose and timing, as an identical manipulation can be adaptive or damaging according to intensity [107,108,110]	Exercise is the best-established intervention for increasing mitochondrial and aerobic capacity in humans. Urolithin A and NAD^+^ precursors show target engagement [94,95,96,97]
**Cellular** **senescence**	Senescent cells carrying combined markers, including p16INK4a, SA-β-gal, and DNA damage foci, accumulate in multiple human tissues with advancing age [2,11,24,115,116] and contribute to aging and selected chronic diseases	Genetic clearance of p16INK4a-positive cells extends lifespan and delays multiple pathologies in mice. Transplantation of senescent cells into young animals causes physical dysfunction. Senolytics improve function and survival in aged mice [114,118,119,120,121]	Transient senescence supports tissue repair, wound healing, and tumor suppression, so indiscriminate elimination carries costs [24]	No single marker defines the senescent state, so quantification requires marker combinations. Human trials of dasatinib plus quercetin remain small; they show feasibility and preliminary target engagement, but no benefit on a primary clinical endpoint has been established [122,123,124]
**Stem-cell** **exhaustion**	Altered abundance, lineage output, clonal selection, and injury response [125]	Restoration of cell-intrinsic or niche pathways improves regeneration in selected models [125,126]	Compartment, niche, clonality, and oncogenic selection [125]	Cell counts are insufficient. Assess lineage output, clonal safety, repair, and organ function [125]
**Altered** **intercellular** **communication**	Altered neural, endocrine, immune, vascular, and extracellular-vesicle signaling [127,134,135]	Parabiosis and exchange studies produce rapid effects without identifying one causal circulating factor [128,132,133]	Dilution, replacement, source, timing, and systemic compensation [131,132]	Defined signals are measurable [129,130,131]
**Chronic** **systemic** **inflammation**	Heterogeneous low-grade activity associated with adverse age-related outcomes [25,137]	IL-1β inhibition establishes disease-specific causality, since canakinumab reduced recurrent cardiovascular events [138]	Cause, location, magnitude, duration, and resolution [25,136]	Markers and drugs are available. Trials must monitor infection, immunity, repair, and function [137,138]
**Gut** **dysbiosis**	Age-associated compositional and functional changes without a universal aged microbiome [140,141]	Transfer alters selected phenotypes in animals. Human causal evidence is limited [142,143,145]	Diet, medication, disease, geography, genotype, residence, and barrier status [140,146]	Microbial functions are modifiable [147,148]. Safety, specificity, and reproducibility constrain broad claims [140]

## Data Availability

No new data were created or analyzed in this study. Data sharing is not applicable to this article.

## Abbreviations

The following abbreviations are used in this manuscript:

**53BP1**	p53-binding protein 1
**AAV**	adeno-associated virus
**acetyl-CoA**	acetyl coenzyme A
**AKT**	AKT serine/threonine kinase (protein kinase B)
**AMPK**	AMP-activated protein kinase
**ATF4**	activating transcription factor 4
**ATG**	autophagy-related protein/gene
**ATG5/ATG12/ATG16L1**	autophagy-related proteins 5, 12, and 16-like 1
**ATM**	ataxia-telangiectasia mutated kinase
**ATP**	adenosine triphosphate
**ATR**	ataxia-telangiectasia and Rad3-related kinase
**BCL-2/BCL2**	B-cell lymphoma 2
**BECN1**	beclin 1
**C/EBPβ**	CCAAT/enhancer-binding protein beta
**CANTOS**	Canakinumab Anti-inflammatory Thrombosis Outcomes Study
**CCL**	C-C motif chemokine ligand
**cFLIP**	cellular FLICE-like inhibitory protein
**cGAS**	cyclic GMP-AMP synthase
**CHK1/CHK2**	checkpoint kinases 1 and 2
**CXCL**	C-X-C motif chemokine ligand
**D+Q**	dasatinib plus quercetin
**DDR**	DNA damage response
**DNA**	deoxyribonucleic acid
**DNMT**	DNA methyltransferase
**DR5**	death receptor 5
**ECM**	extracellular matrix
**eIF2α**	eukaryotic translation initiation factor 2 alpha
**EV**	extracellular vesicle
**FLICE**	FADD-like interleukin-1β-converting enzyme
**FMT**	fecal microbiota transplantation
**FOXO**	forkhead box O transcription factor
**GABARAP**	GABA type A receptor-associated protein
**γH2AX**	phosphorylated histone H2AX
**H3K9me3**	histone H3 lysine 9 trimethylation
**HMGB1**	high-mobility group box 1
**HOPS**	homotypic fusion and protein sorting complex
**HSP90**	heat shock protein 90
**IGF-1**	insulin-like growth factor 1
**IIS**	insulin/IGF-1 signaling
**IL**	interleukin
**IL-1R**	interleukin-1 receptor
**IRAK1**	interleukin-1 receptor-associated kinase 1
**JAK**	Janus kinase
**KLF4**	Krüppel-like factor 4
**LC3**	microtubule-associated protein 1 light chain 3
**LC3-II**	lipidated form of LC3
**LPS**	lipopolysaccharide
**MAPK**	mitogen-activated protein kinase
**mRNA**	messenger RNA
**mtDNA**	mitochondrial DNA
**mTOR**	mechanistic target of rapamycin
**mTORC1**	mechanistic target of rapamycin complex 1
**MYC**	MYC proto-oncogene transcription factor
**NAD^+^**	oxidized nicotinamide adenine dinucleotide
**NF-κB**	nuclear factor kappa B
**NLRP3**	NLR family pyrin domain-containing 3
**OCT4**	octamer-binding transcription factor 4
**OGG1**	8-oxoguanine DNA glycosylase 1
**OSK**	OCT4, SOX2, and KLF4
**p16INK4a**	cyclin-dependent kinase inhibitor 2A isoform p16INK4a
**p21/p21CIP1**	cyclin-dependent kinase inhibitor 1A
**p38 MAPK**	p38 mitogen-activated protein kinase
**p53**	tumor protein p53
**p62/SQSTM1**	sequestosome 1
**PARP**	poly(ADP-ribose) polymerase
**PI3K**	phosphoinositide 3-kinase
**PINK1**	PTEN-induced kinase 1
**RB**	retinoblastoma protein
**RNA**	ribonucleic acid
**ROS**	reactive oxygen species
**SA-β-gal**	senescence-associated beta-galactosidase
**SASP**	senescence-associated secretory phenotype
**SCFA**	short-chain fatty acid
**SES**	socioeconomic status
**SNARE**	soluble NSF attachment protein receptor
**SOX2**	SRY-box transcription factor 2
**SRC**	SRC family tyrosine kinase
**STAT**	signal transducer and activator of transcription
**STING**	stimulator of interferon genes
**TERT**	telomerase reverse transcriptase
**TET**	ten-eleven translocation dioxygenase
**TFEB**	transcription factor EB
**TGF-β**	transforming growth factor beta
**TH10785**	experimental small-molecule OGG1 activator
**TH5487**	experimental small-molecule OGG1 inhibitor
**TNF**	tumor necrosis factor
**TORC1**	target of rapamycin complex 1
**ULK1**	Unc-51-like autophagy activating kinase 1
**UV**	ultraviolet
**VPS34**	vacuolar protein sorting 34
**WNT**	WNT signaling pathway
**YAP-TAZ**	Yes-associated protein (YAP) and transcriptional coactivator with PDZ-binding motif (TAZ)
